# Asthma-related Pediatric Healthcare Utilization after Hospitalization for RSV and Other Viral Lower Respiratory Tract Infections

**DOI:** 10.1093/ofid/ofag402

**Published:** 2026-07-03

**Authors:** Guy Hazan, Mai Ofri, Lital Hertz, Oliver Martyn, David Greenberg

**Affiliations:** Faculty of Health Sciences, Ben-Gurion University, Beer-Sheva, Israel; Pediatric Pulmonary Unit, Saban's Children Hospital, Soroka University Medical Center, Beer-Sheva, Israel; Faculty of Health Sciences, Ben-Gurion University, Beer-Sheva, Israel; Research and Innovation Center, Soroka University Medical Center, Beer-Sheva, Israel; Sanofi Israel Ltd, Vaccines Medical, Petah Tikva, Israel; Sanofi A/S, Vaccines Medical, Copenhagen, Denmark; Faculty of Health Sciences, Ben-Gurion University, Beer-Sheva, Israel; Pediatric Infectious Diseases Unit, Saban's Children Hospital, Soroka University Medical Center, Beer-Sheva, Israel

**Keywords:** pulmonary morbidity, respiratory syncytial virus

## Abstract

**Background:**

Respiratory syncytial virus (RSV) is the leading cause of lower respiratory tract infection (LRTI) in infants. Although influenza, parainfluenza, and human metapneumovirus also contribute to LRTI-related hospitalizations, their comparative population-level healthcare burden is not well defined. This study compared population-level healthcare utilization (HCU) associated with RSV-LRTI and LRTI caused by other respiratory viruses (ORspV-LRTI).

**Methods:**

This nationwide retrospective cohort study used electronic health records from Clalit Health Services (CHS), covering more than 5 million individuals in Israel. Infants born in 2015–2023 and hospitalized before 12 months of age with PCR-confirmed viral LRTI during the RSV season were included. Acute healthcare utilization (HCU) was assessed within 30 days following hospital discharge, and long-term respiratory HCU was evaluated through 6 years of age. HCU was expressed as population-level event rates per 100 000 live births during the study period, and incidence rate ratios (IRRs) were estimated using quasi-Poisson regression.

**Results:**

The cohort included 5822 infants (4951 RSV-LRTI; 871 ORspV-LRTI). RSV-LRTI infants were younger at hospitalization (median 2.7 vs 4.4 months; *P* < .001); Acute HCU was markedly higher in the RSV-LRTI group, including hospitalization duration (IRR = 6.90; 95% CI 4.07–12.6), and anti-asthma medications use (IRR = 4.77–8.04, *P* < .001 for all). Elevated HCU persisted long-term, with higher rates of respiratory medication use, pediatric pulmonologist visits (94.3 vs 16.3 per 100 000; IRR 6.44), and respiratory-related hospitalizations (IRR = 5.39) through 6 years of age. These findings remained consistent in subgroups of younger children and those who experienced more severe disease during the index bronchiolitis hospitalization.

**Conclusions:**

RSV-associated LRTI in infancy is associated with substantially greater population-level HCU than LRTI caused by other respiratory viruses, both acutely and throughout early childhood, supporting prioritization of early-life RSV prevention strategies.

Respiratory syncytial virus (RSV) is the leading cause of lower respiratory tract infections (LRTIs) in infants and young children, responsible for substantial morbidity and a major driver of hospitalization during early childhood [1–4]. Virtually all children are infected with RSV within the first 2 years of life, and a significant proportion, especially those under 12 months, require hospitalization for bronchiolitis, pneumonia, or respiratory distress [1, 5]. Beyond the acute phase, RSV infection during infancy has been associated with long-term respiratory morbidities, including recurrent wheezing and asthma, with implications for ongoing healthcare needs [3, 6–11]. As a result, RSV has been extensively studied in terms of its short- and long-term clinical and economic impact [1, 4, 12]. Recent advances in RSV prevention, including long-acting monoclonal antibodies and maternal immunization, have further underscored the importance of quantifying its full healthcare burden [13–16].

Other respiratory viruses, including influenza, parainfluenza, and human metapneumovirus (hMPV), are likewise well-established causes of LRTIs in young children and contribute meaningfully to hospitalization during early childhood [17]. However, despite their clinical importance, population-level data on healthcare utilization (HCU) associated with these infections, particularly beyond the acute phase, remain limited. Understanding the burden attributable to these viruses, relative to the well-characterized burden of RSV, is essential for contextualizing the true contribution of RSV and informing broader respiratory virus prevention strategies.

These 4 viruses were selected for comparison because they share a biologically coherent profile as causative agents of acute LRTI. Prospective longitudinal studies demonstrate that RSV, influenza, parainfluenza, and hMPV, collectively termed “LRTI-viruses,” dominate during the acute phases of bronchiolitis and pneumonia and decline rapidly within weeks, supporting a direct pathogenic role [18]. This stands in contrast to adenovirus, rhinovirus/enterovirus, and seasonal human coronaviruses, which are commonly detected in both symptomatic children and asymptomatic controls with relatively stable detection over time, suggesting frequent carriage rather than causal involvement in acute LRTI [18]. Focusing on LRTI-viruses therefore enables a more clinically coherent comparison of virus-specific contributions to disease severity, healthcare utilization, and subsequent wheezing morbidity.

To address the gap in comparative burden data, a nationwide retrospective study was conducted to evaluate population-level HCU among infants hospitalized with LRTI due to influenza, parainfluenza, or hMPV, collectively defined as other respiratory virus–associated LRTI (ORspV-LRTI). Given its well-characterized impact, RSV-associated LRTI (RSV-LRTI) was used as the benchmark for comparison, enabling a comprehensive analysis of both acute and long-term HCU across these major respiratory viral infections.

## METHODS

### Study Design and Setting

This retrospective cohort population-based study was based on data from Clalit Health Services (CHS), the largest publicly funded healthcare organization in Israel, covering over 5 million members, approximately 52% of the national population. The study included children born between 2015 and 2023, utilizing CHS's extensive electronic health records. These records provide detailed longitudinal information, including demographic profiles, growth parameters, clinical diagnoses from both hospital and community settings, prescription dispensing data, and laboratory test results [19]. In CHS hospitals, the vast majority of pediatric patients hospitalized for respiratory illness undergo nasal swab testing with a respiratory viral PCR panel, which includes RSV, influenza, human metapneumovirus, parainfluenza, adenovirus, and rhinovirus, as part of routine clinical practice. An exception occurs during weekends (Friday and Saturday in Israel), when patients who are admitted and discharged during these days are typically not swabbed.

All information was anonymized before analysis to ensure patient confidentiality. The study involved secondary analysis of routinely collected health data and was approved by the Institutional Review Board of Soroka University Medical Center (approval number #0330-23-SOR), a CHS-affiliated hospital. As the research was retrospective and based on de-identified data, the requirement for informed consent was waived.

### Study Population

The study population included infants born between 2015 and 2018 in hospitals affiliated with Clalit Health Services (CHS), with complete follow-up through 6 years of age, provided they met the following eligibility criteria:

1. Continuous enrollment in CHS from birth, ensuring the availability of complete medical records.
2. RSV-LRTI group: Infants younger than 12 months hospitalized at a CHS hospital during the RSV season (November 1 to March 31) with a diagnosis of lower respiratory tract infection (LRTI; see Supplementary Table 1), confirmed by ICD-10 codes and a positive RSV polymerase chain reaction (PCR) test [1, 20].
3. ORspV-LRTI group: Infants in the same age range admitted to CHS hospitals during the RSV season, who tested negative for RSV by PCR but positive for one of the following viruses by PCR: influenza, parainfluenza, or human metapneumovirus.
4. Infants were classified as having “high-risk” status for hospitalization if they had 1 or more of the following conditions: prematurity (gestational age ≤37 weeks), small for gestational age, congenital heart disease, neurological disorders, trisomy 21, or chronic pulmonary disease.

Hospitalization episodes occurring outside the RSV season were excluded to ensure epidemiological comparability between groups, as RSV circulates almost exclusively during winter months, and including out-of-season ORspV cases would introduce seasonal confounding from differences in concurrent viral cocirculation and clinical care patterns.

Patients were excluded if they tested positive for more than 1 virus during the same admission, in order to avoid viral coinfections that could obscure attribution of the clinical course to a specific viral pathogen.

#### Data Sources and Organization

De-identified patient-level data were extracted from MDClone, a computerized de-identified database of Clalit Healthcare Services (CHS) electronic medical records. This dataset included information such as date of birth, sex, mode of delivery, gestational age, birth weight, and the presence of multiple births.

#### Study Outcomes

The study assessed acute healthcare utilization (HCU) in children under 12 months of age, defined as HCU occurring within 30 days following hospital discharge.

Acute HCU- included systemic corticosteroid use (OCS), inhaled corticosteroid use (ICS), inhaled short-acting beta-agonist use (SABA), hospital length of stay, number of chest X-rays (CXR), complete blood counts, comprehensive metabolic profiles, and emergency department (ED) visits.

Long-term phase- measured respiratory-related HCU data which were collected until 6 years of age for children hospitalized before their first birthday. This included all acute HCU measures mentioned above, as well as pediatric pulmonologist visits and the number of admissions during this period. ED visits and hospitalizations were classified as respiratory-related based on ICD-10 codes (see Supplementary Table 1).

#### Statistical Analysis

An initial descriptive analysis included calculations of single-variable distributions, measures of central tendency, and dispersion. Further univariate analysis was conducted at the population level to evaluate absolute HCU per 100 000 live births during the study period attributable to each exposure using Quasi Poisson regression for incidence rate ratio (IRR) calculations. Statistical analyses were conducted using R version 4.2.0.

## RESULTS

**Figure 1.**
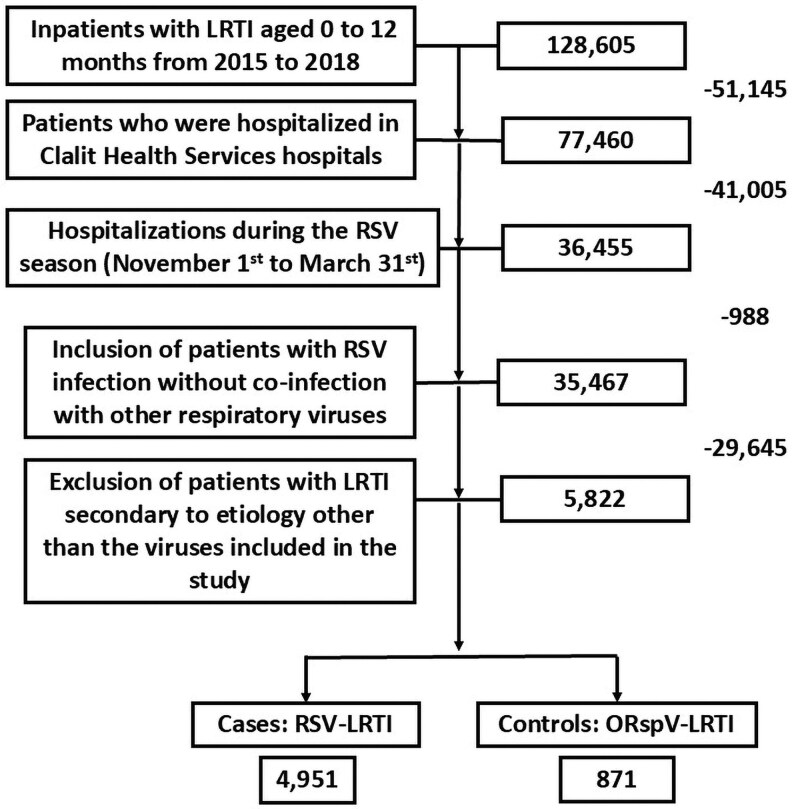
CHS national dataset of infants <12 m hospitalized with LRTI (2015–2018): Cohort inclusion flow. Flowchart depicting the stepwise selection process used to derive the final study cohort. The chart begins with 128 605 inpatients with lower respiratory tract illness (LRTI) aged 0 to 12 m admitted between 2015 and 2018. After excluding 51 145 patients not hospitalized in Clalit Health Services hospitals, 77 460 patients remained. A further 41 005 were excluded as their hospitalization did not occur during RSV season (November 1st to March 31st), leaving 36 455 patients. Exclusion of 988 patients with RSV coinfection with other respiratory viruses yielded 35 467 patients. After excluding 29 645 patients with LRTI secondary to etiologies other than the viruses included in the study, 5822 patients remained. These were divided into 2 final groups: Cases with RSV-LRTI (n = 4951) and Controls with ORspV-LRTI (n = 871).

Between 2015 and 2018, a total of 128 605 CHS-insured infants aged 0–12 months were hospitalized with a diagnosis of LRTI. (Figure 1). Among them, 77 460 (60.2%) were admitted to CHS-operated hospitals, allowing for complete documentation of hospitalization details and follow-up HCU. Furthermore, 41 005 infants whose hospitalizations occurred outside the defined RSV season (November 1 to March 31, see Methods section) were excluded.

An additional 988 cases were excluded due to simultaneous detection of RSV and at least 1 other viral pathogen. Another 29 645 infants were not eligible because their LRTI was attributed to viral causes not included in this study. After applying these exclusion criteria, the final study cohort consisted of 5822 infants. Of these, 4951 infants had a confirmed diagnosis of RSV and were assigned to the RSV-LRTI group, while 871 infants had LRTIs caused by other respiratory viruses and were included in the ORspV-LRTI group. Among the latter, parainfluenza accounted for 315 cases (36%), influenza for 286 cases (33%), and human metapneumovirus for 274 cases (31%).

Table 1 presents the demographic profiles of infants in the RSV-LRTI and ORspV-LRTI groups. Infants hospitalized with RSV-LRTI were notably younger at the time of admission, with a median age of 2.7 months (IQR: 1.43–5.68), compared to 4.39 months (IQR: 1.78–8) in the ORspV-LRTI group, a difference of approximately 1.7 months (*P* < .001). In contrast, no significant differences were observed between the groups in terms of sex distribution (*P* = .5) or ethnicity (*P* = .6). The prevalence of high-risk conditions, including prematurity, congenital heart disease, and chronic pulmonary disorders, was comparable between the 2 groups.

**Table 1.** Demographic and Clinical Variables—Descriptive Analysis With Comparison Between RSV-LRTI to ORspV-LRTI Groups

Characteristic	RSV-LRTI N = 4951	ORspV-LRTI N = 871	*P* Value*
Median (IQR) of Age (months)	2.70 (1.43, 5.68)	4.39 (1.78, 8.00)	<.001
Gender—Male	2734 (55%)	470 (54%)	.5
Ethnicity			.6
Arab	327 (6.9%)	63 (7.5%)	
Jewish	4419 (93%)	782 (93%)	
Cesarean deliveries	793 (20%)	140 (20%)	.9
Mean (±SD) of Birth weight	3176 ± 513	3196 ± 510	.15
High-risk patients^a^	867 (18%)	149 (17%)	.8
Prematurity	359 (7.3%)	60 (6.9%)	.7
Small for gestational age	384 (9.8%)	56 (8.4%)	.3
Chronic heart disease	181 (3.7%)	35 (4.0%)	.6
Neurologic disease	15 (0.3%)	5 (0.6%)	.2
Trisomy 21	18 (0.4%)	3 (0.3%)	>.9
Chronic pulmonary disease	5 (0.1%)	3 (0.3%)	.10

^a^High-risk patients includes: prematurity, small for gestational age, chronic heart disease, neurologic disease, trisomy 21, chronic pulmonary disease.

^*^Wilcoxon rank sum test; Pearson's Chi-squared test; Fisher’s exact test.

Population-level acute healthcare utilization (HCU) rates per 100 000 CHS members within 30 days post-discharge were compared between the RSV-LRTI and ORspV-LRTI groups (Supplementary Table 2). The length of hospital stay was 6.9 times longer in the RSV-LRTI group compared to the ORspV-LRTI group (95% CI: 4.07–12.6; *P* < .001). Use of anti-asthmatic medications within 30 days post-discharge was also significantly higher among children in the RSV-LRTI group. Specifically, OCS purchases were 5.57 times more common (105.3 per 100 000 vs 21 per 100 000; 95% CI: 3.43–9.6; *P* < .001), ICS were 4.77 times more common (60.5 per 100 000 vs 14.08 per 100 000; 95% CI: 2.76–8.82; *P* < .001), and SABAs were 8.04 times more frequently purchased (255.7 per 100 000 vs 35.33 per 100 000; 95% CI: 4.83–14.4; *P* < .001) in the RSV-LRTI group compared to the ORspV-LRTI group.

Table 2 presents a comparison of population-level long-term healthcare utilization rates, expressed per 100 000 CHS members, up to 6 years of age between the RSV-LRTI and ORspV-LRTI groups. Purchases of ICS and SABA were 6.95 and 6.82 times more frequent, respectively, in the RSV-LRTI group compared to the ORspV-LRTI group (95% CI: 3.94–13.4 and 3.88–13.1, respectively, *P* < .001). The number of chest X-rays was also significantly higher in the RSV-LRTI group (469.33 per 100 000 CHS members vs 104.4 per 100 000; IRR = 4.99; 95% CI: 2.52–11.2; *P* < .001).

**Table 2.** Population-Level Long-term Healthcare Utilization Up to 6 Years of Age Rates per 100 000 CHS Members: Comparison Between RSV-LRTI and ORspV-LRTI

Characteristic	RSV-LRTI N = 4951	ORspV-LRTI N = 871	IRR^a^	95% CI^a^	*P* Value
Systemic corticosteroids	268.29	46.98	6.35	3.57, 12.3	<.001
Inhaled corticosteroids	247.65	39.58	6.95	3.94, 13.4	<.001
Short acting beta agonists	314.67	51.23	6.82	3.88, 13.1	<.001
Number of chest X-rays	469.33	104.4	4.99	2.51, 11.2	<.001
Number of pediatric pulmonologist visits	94.33	16.27	6.44	2.73, 18.9	<.001
Number of emergency department visits for respiratory etiology	196.55	27.44	7.96	4.24, 16.8	<.001
Number of hospitalizations for respiratory etiology	80.12	16.51	5.39	2.44, 14.1	<.001

^a^IRR, incidence rate ratio; CI, confidence interval.

Visits to pediatric pulmonologists up to 6 years of age were 6.44 times more common in the RSV-LRTI group (94.33 per 100 000 vs 16.27 per 100 000; 95% CI: 2.73–18.9; *P* < .001). Additionally, the rates of emergency department visits for respiratory causes and respiratory-related hospitalizations up to 6 years of age were 7.96-fold and 5.39-fold higher, respectively, in the RSV-LRTI group compared to the ORspV-LRTI group (*P* < .001).

**Figure 2.**
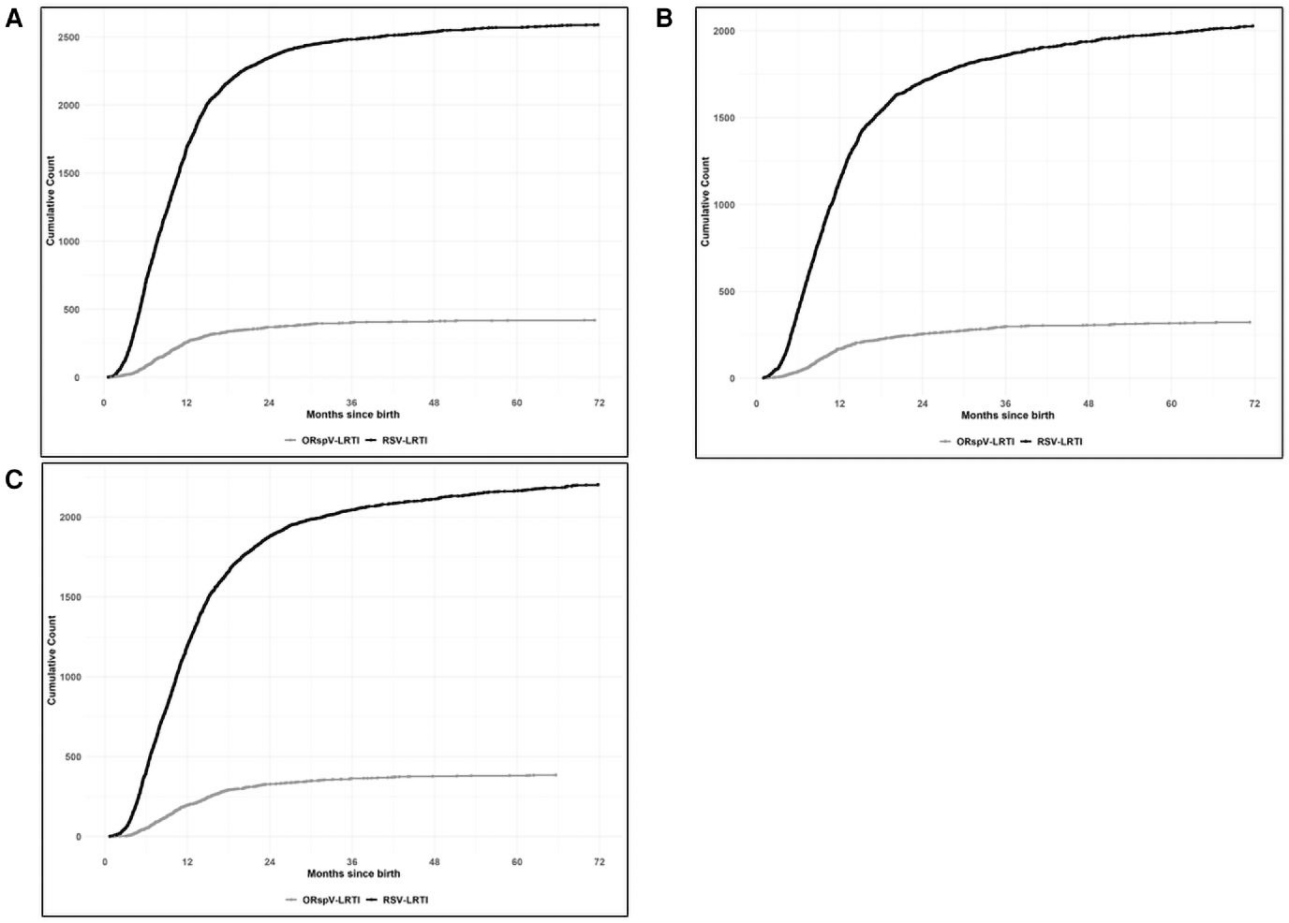
Cumulative healthcare utilization through 6 years of age in children with RSV-LRTI and ORspV-LRTI. *A*. Short-acting β-agonist (SABA) Use. Line graph showing cumulative counts of SABA prescriptions over 72 m since birth in 2 groups of children who experienced early lower respiratory tract illness. The black line representing RSV-LRTI rises steeply between 0 and 24 m, reaching approximately 2400 by 24 m and plateauing near 2600 by 72 m. The gray line representing ORspV-LRTI rises more gradually, reaching approximately 300 by 12 m and plateauing near 425 by 72 m. Cumulative SABA use is markedly higher in the RSV-LRTI group throughout the entire follow-up period. *B*. Inhaled Corticosteroids (ICS) Use. Line graph showing cumulative counts of ICS prescriptions over 72 m since birth in 2 groups of children who experienced early lower respiratory tract illness. The black line representing RSV-LRTI rises steeply between 0 and 24 m, reaching approximately 1650 by 24 m and continuing to rise more gradually to approximately 2025 by 72 m. The gray line representing ORspV-LRTI rises more slowly, reaching approximately 200 by 12 m and plateauing near 340 by 72 m. Cumulative ICS use is substantially higher in the RSV-LRTI group across all time points. *C*. Oral Corticosteroids (OCS) Use. Line graph showing cumulative counts of oral corticosteroid prescriptions over 72 m since birth in 2 groups of children who experienced early lower respiratory tract illness. The black representing RSV-LRTI rises steeply between 0 and 24 m, reaching approximately 1950 by 24 m and continuing to increase gradually to approximately 2200 by 72 m. The gray line representing ORspV-LRTI rises more gradually, reaching approximately 300 by 12 m and plateauing near 400 by 72 m. Cumulative oral corticosteroid use is consistently and substantially higher in the RSV-LRTI group compared to the ORspV-LRTI group throughout the follow-up period.

Cumulative HCU events were examined to assess age-related patterns (Figures 2*A*–*C*). For all anti-asthma medications, including SABA, ICS, and OCS, differences between the RSV-LRTI and ORspV-LRTI groups were driven primarily by events occurring during the first 2 years of life, after which the between-group gap progressively attenuated.

**Figure 3.**
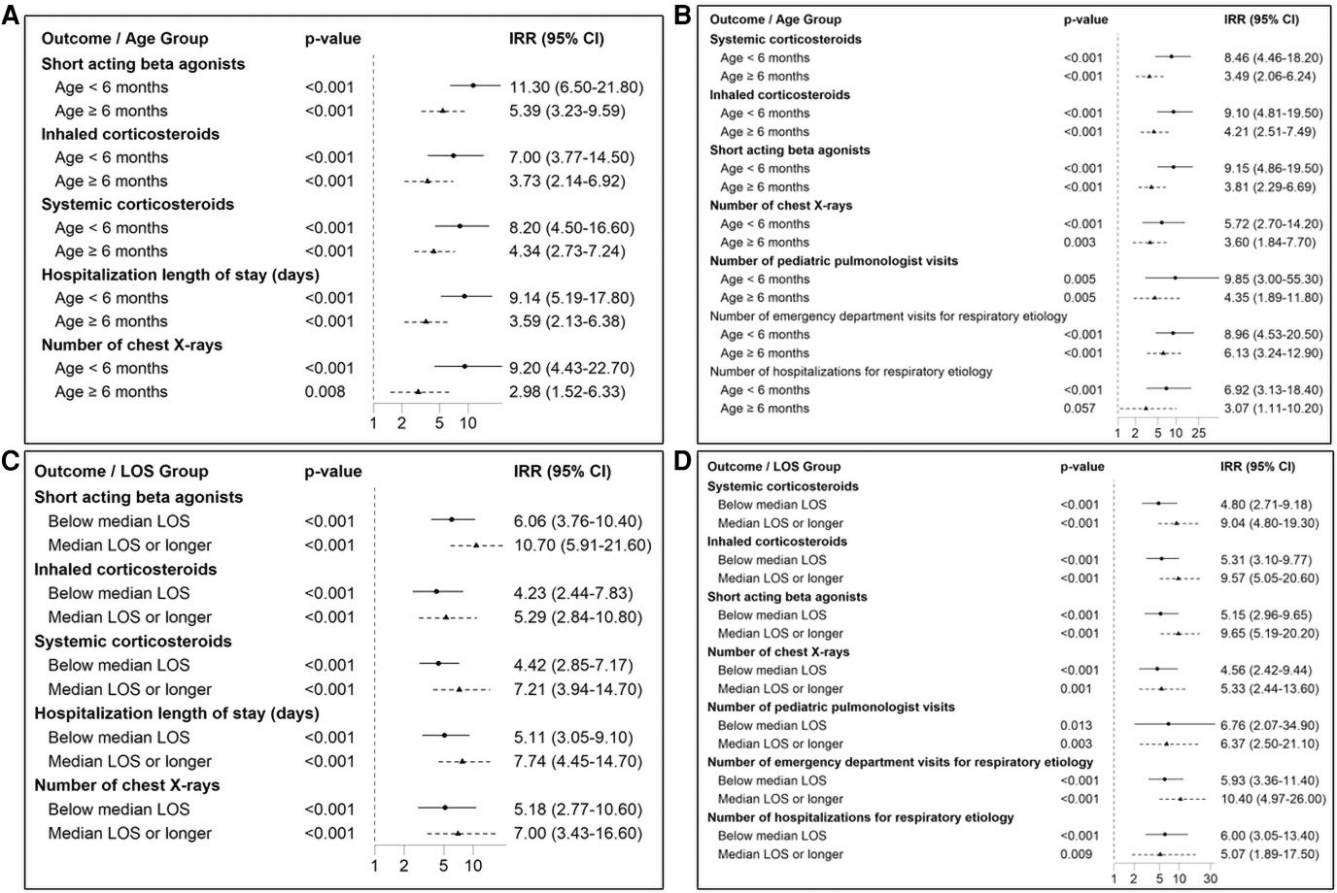
Healthcare utilization for acute and long-term care by age at bronchiolitis episode and length of hospital stay. *A*. Acute Healthcare utilization stratified by age of hospitalization. Forest plot displaying Incidence Rate Ratios (IRR) with 95% confidence intervals for 5 acute healthcare utilization outcomes in children with bronchiolitis, stratified by age group (younger than 6 m shown as solid circles with solid lines; 6 m and older shown as solid triangles with dashed lines). Outcomes include short-acting beta agonists, inhaled corticosteroids, systemic corticosteroids, hospitalization length of stay (days), and number of chest X-rays. All outcomes show statistically significant associations (*P* < .001), except chest X-rays in the older age group (*P* = .008). IRRs are consistently higher in children under 6 m across all outcomes: short-acting beta agonists (11.30 [6.50–21.80] vs 5.39 [3.23–9.59]), inhaled corticosteroids (7.00 [3.77–14.50] vs 3.73 [2.14–6.92]), systemic corticosteroids (8.20 [4.50–16.60] vs 4.34 [2.73–7.24]), hospitalization length of stay (9.14 [5.19–17.80] vs 3.59 [2.13–6.38]), and chest X-rays (9.20 [4.43–22.70] vs 2.98 [1.52–6.33]). The x-axis is on a logarithmic scale ranging from 1 to 10, with a vertical reference line at 1 indicating no effect. *B*. Long-term healthcare utilization stratified by age of hospitalization. Forest plot displaying Incidence Rate Ratios (IRR) with 95% confidence intervals for 7 long-term healthcare utilization outcomes in children with bronchiolitis, stratified by age group (younger than 6 m shown as solid circles with solid lines; 6 m and older shown as solid triangles with dashed lines). Outcomes include systemic corticosteroids, inhaled corticosteroids, short-acting beta agonists, number of chest X-rays, number of pediatric pulmonologist visits, number of emergency department visits for respiratory etiology, and number of hospitalizations for respiratory etiology. IRRs are consistently higher in children under 6 m across all outcomes: systemic corticosteroids (8.46 [4.46–18.20] vs 3.49 [2.06–6.24]), inhaled corticosteroids (9.10 [4.81–19.50] vs 4.21 [2.51–7.49]), short-acting beta agonists (9.15 [4.86–19.50] vs 3.81 [2.29–6.69]), chest X-rays (5.72 [2.70–14.20] vs 3.60 [1.84–7.70]), pediatric pulmonologist visits (9.85 [3.00–55.30] vs 4.35 [1.89–11.80]), emergency department visits (8.96 [4.53–20.50] vs 6.13 [3.24–12.90]), and hospitalizations for respiratory etiology (6.92 [3.13–18.40] vs 3.07 [1.11–10.20]). Most associations are statistically significant (*P* < .001 to *P* = .005), except hospitalizations for respiratory etiology in the older age group (*P* = .057). The x-axis is on a logarithmic scale ranging from 1 to 25, with a vertical reference line at 1 indicating no effect. *C*. Acute Healthcare utilization stratified by length of hospital stay. Forest plot displaying Incidence Rate Ratios (IRR) with 95% confidence intervals for 5 acute healthcare utilization outcomes in children with bronchiolitis, stratified by hospitalization length of stay (LOS) group (below median LOS shown as solid circles with solid lines; median LOS or longer shown as solid triangles with dashed lines). Outcomes include short-acting beta agonists, inhaled corticosteroids, systemic corticosteroids, hospitalization length of stay (days), and number of chest X-rays. All associations are statistically significant (*P* < .001) across both LOS groups. IRRs are consistently higher in the median LOS or longer group for most outcomes: short-acting beta agonists (10.70 [5.91–21.60] vs 6.06 [3.76–10.40]), inhaled corticosteroids (5.29 [2.84–10.80] vs 4.23 [2.44–7.83]), systemic corticosteroids (7.21 [3.94–14.70] vs 4.42 [2.85–7.17]), hospitalization length of stay in days (7.74 [4.45–14.70] vs 5.11 [3.05–9.10]), and chest X-rays (7.00 [3.43–16.60] vs 5.18 [2.77–10.60]). The x-axis is on a logarithmic scale ranging from 1 to 10, with a vertical reference line at 1 indicating no effect. *D.* Long-term Healthcare utilization stratified by length of hospital stay. Forest plot displaying Incidence Rate Ratios (IRR) with 95% confidence intervals for 7 long-term healthcare utilization outcomes in children with bronchiolitis, stratified by hospitalization length of stay (LOS) group (below median LOS shown as solid circles with solid lines; median LOS or longer shown as solid triangles with dashed lines). Outcomes include systemic corticosteroids, inhaled corticosteroids, short-acting beta agonists, number of chest X-rays, number of pediatric pulmonologist visits, number of emergency department visits for respiratory etiology, and number of hospitalizations for respiratory etiology. All associations are statistically significant across both LOS groups (*P* < .001 to *P* = .013). IRRs are consistently higher in the median LOS or longer group for most outcomes: systemic corticosteroids (9.04 [4.80–19.30] vs 4.80 [2.71–9.18]), inhaled corticosteroids (9.57 [5.05–20.60] vs 5.31 [3.10–9.77]), short-acting beta agonists (9.65 [5.19–20.20] vs 5.15 [2.96–9.65]), chest X-rays (5.33 [2.44–13.60] vs 4.56 [2.42–9.44]), pediatric pulmonologist visits (6.76 [2.07–34.90] vs 6.37 [2.50–21.10]), emergency department visits for respiratory etiology (10.40 [4.97–26.00] vs 5.93 [3.36–11.40]), and hospitalizations for respiratory etiology (6.00 [3.05–13.40] vs 5.07 [1.89–17.50]). The x-axis is on a logarithmic scale ranging from 1 to 30, with a vertical reference line at 1 indicating no effect.

Next, analyses of HCU were stratified according to age at hospitalization and median length of stay (Figures 3*A*–*D*). Stratification by age at the time of the bronchiolitis episode demonstrated consistently higher acute HCU among infants hospitalized before 6 months of age compared with those hospitalized at 6 months of age or older across all evaluated outcomes (Figure 3*A*). Children younger than 6 months had significantly higher rates of SABA purchase (IRR 11.30, 95% CI 6.50–21.80 vs IRR 5.39, 95% CI 3.23–9.59) and OCS purchase (IRR 8.20, 95% CI 4.50–16.60 vs IRR 4.34, 95% CI 2.73–7.24). Similarly, hospitalization length of stay was markedly longer among younger infants (IRR 9.14, 95% CI 5.19–17.80 vs IRR 3.59, 95% CI 2.13–6.38).

This pattern persisted across long-term outcomes up to the age of 6 (Figure 3*B*). Rates of OCS purchase (IRR 8.46, 95% CI 4.46–18.20 vs IRR 3.49, 95% CI 2.06–6.24), ICS purchase (IRR 9.10, 95% CI 4.81–19.50 vs IRR 4.21, 95% CI 2.51–7.49), and SABA purchase (IRR 9.15, 95% CI 4.86–19.50 vs IRR 3.81, 95% CI 2.29–6.69) were all substantially higher in the younger age group. Chest X-rays (IRR 5.72, 95% CI 2.70–14.20 vs IRR 3.60, 95% CI 1.84–7.70) and pediatric pulmonologist visits (IRR 9.85, 95% CI 3.00–55.30 vs IRR 4.35, 95% CI 1.89–11.80) followed the same trend. Respiratory-related rehospitalizations were significantly higher among infants younger than 6 months (IRR 6.92, 95% CI 3.13–18.40; *P* < .001), while the association in the older group did not reach statistical significance (IRR 3.07, 95% CI 1.11–10.20; *P* = .057).

When stratified by length of hospital stay, children with a median LOS or longer had higher rates of acute HCU across all outcomes compared with those below the median (Figure 3*C*). SABA purchase showed the largest absolute difference between LOS groups (IRR 10.70, 95% CI 5.91–21.60 vs IRR 6.06, 95% CI 3.76–10.40), followed by OCS purchase (IRR 7.21, 95% CI 3.94–14.70 vs IRR 4.42, 95% CI 2.85–7.17). Interestingly, longer index hospitalization was similarly associated with higher long-term HCU across most outcomes (Figure 3*D*). Children with median LOS or longer had markedly higher rates of SABA purchase (IRR 9.65, 95% CI 5.19–20.20 vs IRR 5.15, 95% CI 2.96–9.65), ICS purchase (IRR 9.57, 95% CI 5.05–20.60 vs IRR 5.31, 95% CI 3.10–9.77), and OCS purchase (IRR 9.04, 95% CI 4.80–19.30 vs IRR 4.80, 95% CI 2.71–9.18). Emergency department visits for respiratory etiology were also substantially higher in the longer LOS group (IRR 10.40, 95% CI 4.97–26.00 vs IRR 5.93, 95% CI 3.36–11.40).

## DISCUSSION

This large-scale retrospective study provides a comprehensive population-level evaluation of healthcare utilization among infants hospitalized with RSV-associated lower respiratory tract infection (RSV-LRTI) compared with those hospitalized with influenza-, parainfluenza-, or human metapneumovirus–associated LRTI (other respiratory virus LRTI; ORspV-LRTI). By leveraging comprehensive longitudinal electronic health records from Clalit Health Services, covering over 52% of the Israeli population, the study captures both acute and long-term HCU outcomes across an 8-year period spanning multiple RSV seasons. The findings consistently show that infants with RSV-LRTI had markedly higher HCU compared to those with ORspV-LRTIs, including longer hospital stays, increased use of respiratory medications (systemic corticosteroids, inhaled corticosteroids, and short-acting beta-agonists), and more frequent chest radiography during the acute phase. Notably, these differences extended well beyond the initial hospitalization, with the RSV-LRTI group exhibiting significantly higher rates of pediatric pulmonologist visits, respiratory-related emergency department visits, hospitalizations, and respiratory medication use up to 6 years of age. Furthermore, the observed associations remained robust in subgroup analyses of children hospitalized before 6 months of age and those with a longer duration of the index hospitalization. These results highlight the sustained healthcare burden associated with RSV infection in infancy in the population level.

Although several studies have compared healthcare utilization associated with RSV and other respiratory viruses, data on the long-term, up to 6 years of age, remain limited [21]. The findings of the present research align with and extend existing literature, adding critical longitudinal perspective on the prolonged burden of RSV—particularly in the context of a clinically active comparator group of infants hospitalized with other major respiratory viruses, rather than uninfected controls. Importantly, the RSV-LRTI group was younger at hospitalization (median 2.7 vs 4.4 months), consistent with the known predilection of RSV for the youngest infants. While this age difference may partially contribute to observed HCU differences, it also reflects a core feature of RSV epidemiology and underscores the vulnerability of very young infants to the downstream consequences of RSV infection. . A comprehensive systematic review and meta-analysis [17] quantified the clinical spectrum of 4 major respiratory viruses: RSV, influenza, parainfluenza, and human metapneumovirus in preschool children. Among these, RSV was the most symptomatic, with 71.8% of test-positive children under 2 years exhibiting symptoms, and it carried the highest risk of progressing to severe disease. Notably, the likelihood of hospitalization and very severe illness was highest in younger age groups and in low- and middle-income countries.

Supporting these findings, Australian cohort study [22] analyzed over 210 000 hospitalized children under 2 years with laboratory-confirmed viral infections. RSV was associated with the highest rates of hospitalization and clinical severity compared to other viruses. Infants with RSV required respiratory support earlier in the course of illness and had an increased risk of readmission within 30 days due to acute LRTI.

The association between RSV infection in infancy and subsequent asthma inception is well established in the literature [10, 23, 24]; however, whether this relationship is causal remains unresolved. One hypothesis suggests that RSV infection in early life may alter airway structure or inflammatory responses [25], thereby contributing directly to asthma inception, whereas an alternative explanation proposes that severe RSV infection serves primarily as a marker of underlying susceptibility to asthma rather than a causal factor [19, 26–28]. Although the present study cannot resolve this debate, it strengthens the observed association between RSV-related LRTI and later asthma outcomes by demonstrating that this relationship persists even when the reference group comprises infants hospitalized with lower respiratory tract infections caused by other respiratory viruses.

Subgroup analyses further delineated the populations at greatest risk for sustained healthcare burden. Stratification by age at hospitalization demonstrated that infants younger than 6 months experienced markedly higher HCU compared with those hospitalized at 6 months or older, both acutely and through 6 years of age. These findings align with evidence that age at RSV infection modulates long-term respiratory morbidity, as the first year of life represents a critical window of lung and immune development during which RSV infection may predispose to subsequent wheezing and asthma inception [10, 29]. Similarly, stratification by hospitalization length of stay revealed a dose-response pattern, whereby children with a longer index admission, a proxy for acute illness severity, demonstrated substantially greater long-term HCU compared with shorter-stay counterparts. Taken together, these findings suggest that both younger age at infection and greater initial disease severity serve as compounding markers of long-term respiratory morbidity, reinforcing the importance of early RSV prevention, particularly through broad immunization strategies targeting infants in the first months of life. A key incremental contribution of this study is the longitudinal follow-up of HCU through 6 years of age, extending well beyond the acute phase and the first 2 years of life that have been the focus of most prior research. This long-term perspective is particularly valuable for informing cost-effectiveness analyses of RSV prophylaxis, as it captures the full downstream burden of respiratory medication use, specialist visits, and recurrent hospitalizations that must be weighed against the cost of preventive interventions.

The present study has several limitations that should be considered when interpreting the findings. First, retrospective design relies on electronic health records, which may be subject to coding inaccuracies or misclassification of diagnoses and healthcare utilization events. Although PCR confirmation of viral etiology enhances diagnostic accuracy, not all hospitalized children had PCR tests, and it is possible that some data were missing. Second, while the RSV group was used as a reference for comparison, differences in age at hospitalization and potential unmeasured confounders, such as socioeconomic status or environmental exposures, may have influenced both the likelihood of infection and subsequent utilization patterns. However, when the underlying disease and other demographic data were compared, no differences were found between groups. Despite these limitations, the study's large sample size and rich longitudinal follow-up provide robust and meaningful insights into the differential healthcare burden associated with RSV and other respiratory viral infections in early life.

The present study underscores the population-level burden of RSV during the first 12 months of life, demonstrating a greater impact compared with the 3 other major respiratory viruses considered as a group. Moreover, it highlights the critical needs to prioritize RSV prevention strategies in early infancy. The novelty of this study lies in its population-level comparison of RSV burden with that of other respiratory viruses, focusing on healthcare utilization at the population level rather than on per-patient risk, thereby enabling conclusions that are directly relevant to healthcare planning and policy decisions. By demonstrating that RSV-associated LRTI leads to substantially higher HCU not only during the acute illness but also over several years of follow-up, this study provides important real-world evidence on the prolonged burden of RSV. Taken together, these data support the implementation of broad, population-level RSV immunization programs to reduce early-life respiratory morbidity and its long-term sequelae, including repeated healthcare utilization through school age. Importantly, this study adds unique longitudinal, population-level data to a field where most prior research has focused on per-patient, short-term outcomes, offering a more complete picture of RSV true burden in early childhood and strengthening the evidence base for policy prioritization of RSV prevention.

## Supplementary Material

ofag402_Supplementary_Data

## Supplementary Data

Supplementary materials are available at *Open Forum Infectious Diseases* online. Consisting of data provided by the authors to benefit the reader, the posted materials are not copyedited and are the sole responsibility of the authors, so questions or comments should be addressed to the corresponding author.
