# Supplementary material for: Asthma-related Pediatric Healthcare Utilization after Hospitalization for RSV and Other Viral Lower Respiratory Tract Infections
Source: Open Forum Infect Dis. 2026 Jul 3;13(7):ofag402. doi: 10.1093/ofid/ofag402 (PMC13358292; doi:10.1093/ofid/ofag402)
Supplement: ofag402_Supplementary_Data [file ofag402_supplementary_data.docx]

**Table 1S – ICD-10 codes for respiratory-related symptoms:**

| **Diagnoses** | **ICD10 code** |
| --- | --- |
| **Pneumonia** | J09-J18 |
| **Acute bronchiolitis** | J20-J21 |
| **Asthma** | J44-J45 |
| **Cough** | R05 |
| **Wheezing** | R06.2 |
| **Hypoxemia** | R09.02 |

**Table 2S. Population-Level Acute Healthcare Utilization Rates per 100,000 CHS Members Within 30 Days Post-Discharge: Comparison Between RSV-LRTI and ORspV-LRTI**

| **Characteristic** | **RSV-LRTI**  **N = 4,951** | **ORspV-LRTI**  **N = 871** | **IRR***^1^* | **95% CI***^1^* | **p-value** |  |
| --- | --- | --- | --- | --- | --- | --- |
| Systemic corticosteroids | 105.25 | 21 | 5.57 | 3.43, 9.60 | <0.001 |  |
| Inhaled corticosteroids | 60.46 | 14.08 | 4.77 | 2.76, 8.82 | <0.001 |  |
| Short acting beta agonists | 255.67 | 35.33 | 8.04 | 4.83, 14.4 | <0.001 |  |
| Hospitalization length of stay (days) | 2,193.54 | 353.19 | 6.90 | 4.07, 12.6 | <0.001 |  |
| Number of chest X-rays | 598.62 | 105.62 | 6.30 | 3.24, 13.8 | <0.001 |  |
| *^1^* IRR = Incidence Rate Ratio, CI = Confidence Interval | | | | | | |
